# Perioperative outcome of left atrial appendage amputation in coronary artery bypass grafting

**DOI:** 10.1007/s00392-024-02529-9

**Published:** 2024-09-02

**Authors:** Mustafa Gerçek, Tomislav Skuljevic, Jochen Börgermann, Jan Gummert, Muhammed Gerçek

**Affiliations:** 1 Clinic for Cardiac Surgery and Pediatric Cardiac Surgery, Heart Center Duisburg, Gerrickstraße 21, 47137 Duisburg, Germany; 2 Clinic for Thoracic and Cardiovascular Surgery, Herz- Und Diabeteszentrum NRW, Georgstraße 11, 32545 Bad Oeynhausen, Germany; 3 Clinic for General and Interventional Cardiology/Angiology, Herz- Und Diabeteszentrum NRW, Ruhr-Universität Bochum, Med. Fakultät OWL (Universität Bielefeld), Georgstraße 11, 32545 Bad Oeynhausen, Germany

**Keywords:** CABG, LAA amputation, Cardiac surgery, Stroke, Revascularization

## Abstract

**Background:**

Left atrial appendage (LAA) amputation performed alongside cardiac surgery has become an increasingly established procedure to reduce stroke risk in patients with atrial fibrillation. As the recommendation levels for LAA amputation continue to rise, ample evidence assessing its perioperative safety and risk factors is of utmost interest.

**Methods:**

All patients who underwent isolated coronary artery bypass grafting (CABG) between 2018 and 2021 at two high-volume centers were retrospectively included in the study. Patients were divided into two groups—the CABG and CABG + LAA groups—based on whether they underwent concomitant LAA amputation. Propensity score matching (PS matching) was applied to ensure comparability between the groups. The primary endpoint was defined as a composite outcome comprising of all-cause mortality, stroke, and reoperation. Secondary endpoints included the components of the primary endpoint, perioperative outcome parameters, transfusion rates, and laboratory parameters.

**Results:**

A total of 3904 patients were included with 3038 and 866 in the CABG and CABG + LAA group, respectively. After PS matching each group consisted of 856 patients. The primary endpoint showed no significant differences between the CABG and CABG + LAA group (7.0% vs. 6.5% (OR 0.9 95% CI [0.64; 1.35], *p* = 0.70)). Similarly, there were no notable differences in the individual components of the composite endpoint: all-cause mortality (*p* = 0.84), stroke (*p* = 0.74), and reoperation (*p* = 0.50). Subgroup results did not show any relevant dissimilarity.

**Conclusion:**

The concomitant performance of LAA amputation is not associated with worse in-hospital outcomes, as measured by the composite endpoint of all-cause mortality, stroke, and reoperation.

**Graphical abstract:**

Perioperative outcome of left atrial appendage amputation in coronary artery bypass grafting. 95% CI, 95% confidence intervals; CABG, coronary artery bypass grafting; EF, left ventricular ejection fraction; LAA, left atrial appendage amputation; OR, odds ratio

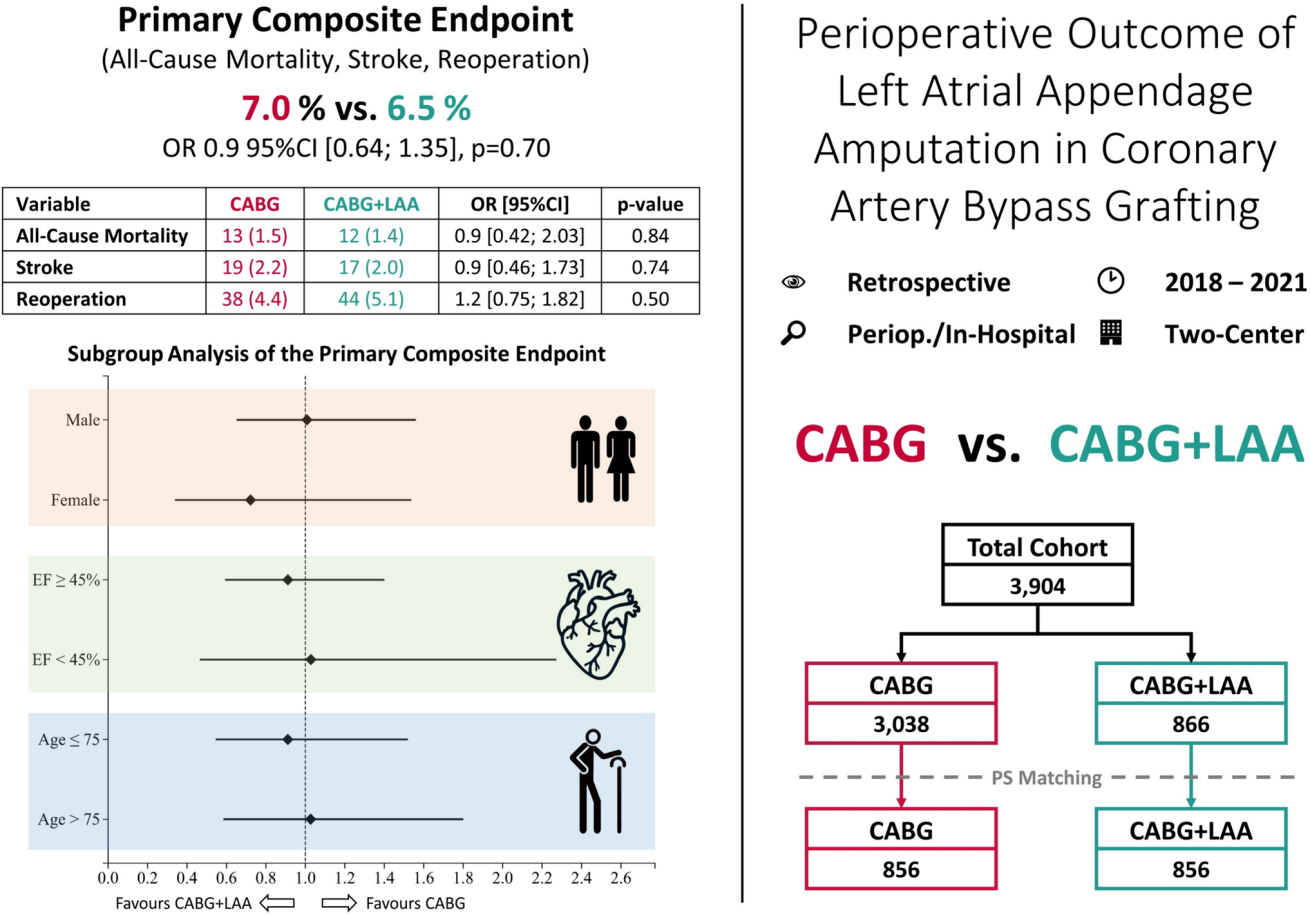

**Supplementary Information:**

The online version contains supplementary material available at 10.1007/s00392-024-02529-9.

## Introduction

The left atrial appendage (LAA) is a significant focus in cardiac surgery, interventional cardiology, electrophysiology, and ablation [1–3]. It has been identified as the main source of stroke-causing thrombi in patients with atrial fibrillation (AF) and serves as a key target in electrophysiologic ablation therapy [1, 4]. Therefore, the occlusion or amputation of the LAA has become a recommended approach for patients with atrial fibrillation. This approach complements the gold standard therapy of effective anticoagulation to prevent stroke in patients at risk [5, 6]. Following the LAAOS III trial, which proved the efficacy of LAA amputation in a prospective 5-year follow-up [7], the recommendation to perform LAA amputation was rapidly elevated to IIa [8].

Coronary artery bypass grafting (CABG) is the most frequently performed procedure in cardiac surgery [9], largely due to the high prevalence of coronary artery disease (CAD), particularly in affluent societies with increasing rates of metabolic diseases [10]. Consequently, the majority of concomitant execution of LAA amputations are likely to occur in CABG surgery. Surgically, the LAA is easily accessible via median sternotomy in conventional cardiac surgery or even anterolateral thoracotomy in minimally invasive approaches [3, 11]. However, challenging surgical scenarios or concerns about increasing the risk of perioperative bleeding and subsequent complications may lead surgeons to avoid concomitant procedures, especially those involving penetration of cardiac chambers, such as LAA amputation.

Balancing a prophylactic procedure to reduce the long-term stroke risk while maintaining the perioperative surgical safety is crucial. Nevertheless, as recent evidence on the long-term benefits of LAA amputation promotes a broader adoption of this procedure in cardiac surgery, evaluating the procedures perioperative safety and identifying subgroups at risk are of utmost importance. These are essential for developing and recommending a safe and targeted therapeutic strategy.

## Patients and methods

### Ethical statement

Approval, including patient consent waiver, was obtained from the local ethics committee of the Ruhr University Bochum (No: 2023_1093; Date: 11.07.2023) and the ethics committee of the Ärztekammer Nordrhein (No: 88/2023; Date: 12.04.2023). All patients in the CABG + LAA cohort received a thoroughly dedicated patient information concerning the LAA amputation. Furthermore, the study was conducted in accordance with the ethical standards laid down in the 1964 Declaration of Helsinki and its later amendments.

### Patient recruitment and follow-up

All patients undergoing elective isolated coronary artery bypass grafting (CABG) between January 2018 and December 2021 at two centers—Heart Center Duisburg, Duisburg, Germany and Herz- und Diabeteszentrum NRW, Bad Oeynhausen, Germany—were retrospectively enrolled. Preoperative exclusion criteria included any concomitant procedure on valves or the aorta. Necessary condition to execute concomitant LAA amputation was preoperative atrial fibrillation (paroxysmal, persistent, and permanent). The recruited patients were divided into two groups: Those with isolated CABG (CABG) and those with CABG with concomitant LAA amputation (CABG + LAA).

### Surgical technique

The surgical procedure was performed through a median sternotomy, and well-established grafts such as the left and/or right internal mammary artery, radial artery, and saphenous vein. In the CABG + LAA cohort, the accessibility of the LAA was assessed by the surgeon after pericardial exposure. LAA amputation was typically performed using ligation, LAA resection, and double continuous suture, while a small, unmeasured minority were performed by clipping or stapling. Transesophageal echocardiography was employed to check for pre-existing thrombus formation and to detect a residual appendage after amputation. Given that the use of grafts, number of anastomosis, manipulation of the aorta, and the operative technique (conventional/off-pump) varied within the cohort, these operative parameters were included in the matching.

### Outcome

The primary outcome was defined as a composite endpoint of all-cause mortality, stroke and reoperation during the in-hospital period. The parameters forming the composite endpoints also served as secondary endpoints. Additionally, perioperative complications (need for dialysis, invasive coronary angiography (ICA), intra-aortic balloon-pump (IABP), extracorporeal life support (ECLS), cardiopulmonary resuscitation (CPR), pericardial effusion, intensive care unit (ICU) stay, and transfusions (packed red blood cells (PRBC), fresh frozen plasma (FFP)), along with biochemical markers (creatinine kinase (CK), creatinine kinase isoenzyme MB (CK-MB), troponin, creatinine)) were assessed as further secondary endpoints. The endpoint stroke was defined as either confirmation of stroke through cerebral imaging (computed tomography or MRI) or the presence of unequivocal neurological impairment, such as hemiplegia. The need for ICA was determined by the patient’s condition, including hemodynamic instability, echocardiographic deterioration, changes in electrocardiography or rhythm prompting the suspicion of new myocardial ischemia, but it does not represent proof of anastomosis failure. Reoperation is defined by any condition, such as bleeding, cardiac tamponade, or myocardial ischemia which led to the decision to perform a reoperation.

To assess the impact of subgroup differences on the primary endpoint and its components, subgroups based on gender (male, female), contractility (left ventricular ejection fraction (EF) ≥ 45%, EF < 45%), and age (≤75 years, >75 years) were examined.

### Statistical analysis

Statistical analysis was performed using the SPSS-Software (Version 28, IBM, New York, NY, USA) and R (Version 4.2.2, R Core Team, Vienna, Austria). Categorical variables are presented as absolute and relative frequencies, while continuous variables are illustrated as means with standard deviations. Due to non-randomized retrospective group selection, we used 1:1 PS matching on CABG and CABG + LAA cohorts utilizing nearest neighbor matching with a caliper of 0.2 by all available baseline characteristics, medication, echocardiography, and operative parameters (Table 1). The balance of baseline covariates before and after matching was assessed by computing the standardized mean difference (balance achieved if <|0.1|) [12, 13]. Endpoint analysis was conducted using the chi-squared test or Fisher’s exact test for categorical variables, and unpaired *t* tests for continuous variables. In the matched cohorts, continuous variables were compared using paired *t* tests. The analysis is presented in the matched cohorts, whereas the results in the unmatched cohorts are provided in the supplements.

**Table 1:** Baseline characteristics of the total cohort, the unmatched and the propensity score matched cohorts

Variable	Unmatched cohorts					Propensity score matched cohorts				
	CABG *n* (%)	CABG + LAA *n* (%)	OR [95% CI]	SMD	*p* value	CABG *n* (%)	CABG + LAA *n* (%)	OR [95% CI]	SMD	*p* value
	3038 (100)	866 (100)				856 (100)	856 (100)			
*Baseline parameter*										
Age	66.9 ± 9.8	70.9 ± 7.8	−4.67; −3.26	0.51	<0.01	70.9 ± 8.66	70.78 ± 7.7	−0.59; 0.78	−0.01	0.78
Gender (female)	564 (18.6)	179 (20.7)	1.1 [0.95; 1.38]	0.05	0.16	169 (19.7)	174 (20.3)	1.0 [0.82; 1.31]	0.01	0.76
Height (cm)	173.0 ± 9.0	172.6 ± 8.7	−0.29; 1.06	−0.05	0.26	172.4 ± 9.2	172.6 ± 8.8	−1.09; 0.63	0.03	0.61
Weight (kg)	89.3 ± 21.7	85.7 ± 19.5	2.01; 5.21	−0.19	<0.01	85.9 ± 18.4	85.8 ± 19.5	−1.60; 1.85	−0.007	0.88
BMI (kg/m^2^ BSA)	29.8 ± 6.8	28.7 ± 5.8	0.62; 1.62	−0.19	<0.01	28.8 ± 5.4	28.7 ± 5.8	−0.39; 0.62	−0.02	0.64
Center	2022 (66.6)	773 (89.23)	4.2 [3.33; 5.25]	0.73	<0.01	769 (89.8)	763 (89.1)	0.9 [0.68; 1.26]	−0.02	0.64
DM	1142 (37.6)	385 (44.5)	1.3 [1.14; 1.55]	0.14	<0.01	386 (45.1)	380 (44.4)	1.0 [0.80; 1.18]	−0.01	0.77
HLP	2783 (91.6)	819 (94.6)	1.6 [1.16; 2.20]	0.13	<0.01	810 (94.6)	809 (94.5)	1.0 [0.64; 1.48]	−0.01	0.92
AHT	2748 (90.5)	818 (94.5)	1.8 [1.31; 2.46]	0.18	<0.01	810 (94.6)	808 (94.4)	1.0 [0.63; 1.45]	−0.01	0.83
COPD	379 (12.5)	105 (12.1)	1.0 [0.77; 1.22]	−0.01	0.78	101 (11.8)	104 (12.2)	1.0 [0.77; 1.38]	0.01	0.82
pAD	351 (11.6)	139 (16.1)	1.5 [1.18; 1.81]	0.12	<0.01	140 (16.4)	138 (16.1)	1.0 [0.76; 1.27]	−0.01	0.90
cAD	302 (9.9)	129 (14.9)	1.6 [1.27; 1.98]	0.14	<0.01	121 (14.1)	125 (14.6)	1.0 [0.79; 1.36]	0.01	0.78
NYHA (class)	2.3 ± 0.8	2.3 ± 0.7	−0.05; 0.07	−0.02	0.67	2.3 ± 0.8	2.3 ± 0.7	−0.07; 0.07	−0.008	0.87
CCS (class)	1.8 ± 1.1	1.5 ± 1.1	0.20; 0.37	−0.26	<0.01	1.5 ± 1.2	1.5 ± 1.1	−0.09; 0.12	−0.02	0.76
Preop. Pacemaker	90 (3)	42 (4.9)	1.7 [1.15; 2.43]	0.09	<0.01	41 (4.8)	41 (4.8)	1.0 [0.64; 1.56]	0	>0.99
*Medication*										
ASA	2568 (84.5)	656 (75.8)	0.6 [0.48; 0.69]	−0.21	<0.01	652 (76.2)	654 (76.4)	1.0 [0.81; 1.27]	0.01	0.91
P2Y2 inhibitor	349 (11.5)	115 (13.9)	1.2 [0.94; 1.48]	0.05	0.15	107 (12.5)	112 (13.1)	1.1 [0.79; 1.40]	0.01	0.72
CCB	692 (22.8)	214 (24.7)	1.1 [0.93; 1.33]	0.05	0.24	212 (24.8)	210 (24.5)	1.0 [0.79; 1.23]	−0.01	0.91
Statins	2428 (79.9)	692 (79.9)	1.0 [0.83; 1.21]	−0.00	>0.99	683 (79.8)	684 (79.9)	1.0 [0.80; 1.28]	0.003	0.95
ACEs	1331 (43.8)	400 (46.2)	1.1 [0.95; 1.28]	0.05	0.21	395 (46.1)	396 (46.3)	1.0 [0.83; 1.21]	0.002	0.96
ARB	1060 (34.9)	330 (38.1)	1.2 [0.98; 1.34]	0.07	0.08	333 (38.9)	325 (38.0)	1.0 [0.79; 1.17]	−0.02	0.69
Beta blockers	1843 (60.7)	577 (66.6)	1.3 [1.10; 1.52]	0.13	<0.01	586 (68.5)	568 (66.4)	0.9 [0.74; 1.11]	−0.05	0.35
MRA	208 (6.8)	45 (5.2)	0.8 [0.54; 1.04]	−0.07	0.08	42 (4.9)	44 (5.1)	1.1 [0.68; 1.62]	0.01	0.83
Diuretics	1026 (33.8)	344 (39.7)	1.3 [1.11; 1.51]	0.12	<0.01	348 (40.7)	337 (39.4)	1.0 [0.78; 1.15]	−0.03	0.59
*Echocardiography*										
Preop. EF (%)	53.0 ± 9.5	52.9 ± 9.5	−0.69; 0.75	−0.00	0.93	52.9 ± 9.7	52.9 ± 9.5	−0.95; 0.83	0.005	0.91
Preop. AR (grade)	0.1 ± 0.4	0.2 ± 0.5	−0.10; −0.04	0.14	<0.01	0.2 ± 0.5	0.2 ± 0.5	−0.06; 0.03	0.03	0.56
Preop. AS (grade)	0.0 ± 0.3	0.1 ± 0.3	−0.05; 0:00	0.07	0.08	0.1 ± 0.3	0.1 ± 0.3	−0.04; 0.03	−	>0.99
Preop. MR (grade)	0.4 ± 0.6	0.5 ± 0.7	−0.18; −0.09	0.19	<0.01	0.4 ± 0.7	0.5 ± 0.7	−0.10; 0.03	0.04	0.35
Preop. MS (grade)	0.0 ± 0.1	0.0 ± 0.1	−0.01; 0:00	−0.00	0.95	0.0 ± 0.1	0.0 ± 0.1	−0.01; 0.00	0	>0.99
Preop. TR (grade)	0.1 ± 0.4	0.2 ± 0.5	−0.13; −0.07	0.19	<0.01	0.2 ± 0.5	0.2 ± 0.5	−0.07; 0.02	0.03	0.48
Preop. TS (grade)	0.0 ± 0.0	0.0 ± 0.0	−0.01; 0.00	−0.03	0.45	0.0 ± 0.0	0.0 ± 0.0	–	0	–
*Operative parameters*										
Operation time (min)	212.5 ± 55.5	210.7 ± 50.8	−2.28; 5.95	0.00	0.36	211.7 ± 78.8	211.0 ± 50.9	−5.69; 7.05	−0.01	0.83
OPCAB	2493 (82.1)	727 (84.0)	1.1 [0.93; 1.40]	0.05	0.20	701 (81.9)	717 (83.8)	1.1 [0.89; 1.47]	0.05	0.31
No aortic touch	959 (31.6)	188 (21.7)	0.6 [0.50; 0.72]	−0.24	<0.01	204 (23.8)	187 (21.9)	0.9 [0.71; 1.12]	−0.05	0.33
Clampless anastomosis										
AAP	968 (31.9)	400 (46.2)	1.8 [1.57; 2.14]	0.29	<0.01	362 (42.3)	392 (45.8)	1.2 [0.95; 1.40]	0.07	0.14
AoSD	521 (17.1)	123 (14.2)	0.8 [0.65; 0.99]	−0.08	0.039	117 (13.7)	122 (14.3)	1.1 [0.80; 1.38]	0.02	0.73
BIMA	847 (27.9)	216 (24.9)	0.9 [0.72; 1.02]	−0.07	0.09	216 (25.2)	211 (24.7)	1.0 [0.78; 1.21]	−0.01	0.78
Radialis	53 (1.7)	3 (0.4)	0.2 [0.06; 0.63]	−0.24	<0.01	4 (0.5)	3 (0.4)	0.8 [0.17; 3.36]	−0.02	0.71
TAR	780 (25.7)	130 (15.0)	0.5 [0.42; 0.63]	−0.30	<0.01	142 (16.6)	129 (15.1)	0.9 [0.69; 1.16]	−0.04	0.39
Distal anast. (no)	2.9 ± 0.9	3.0 ± 0.8	−0.24; −0.12	0.23	<0.01	3.0 ± 0.8	3.0 ± 0.8	−0.08; 0.07	−0.003	0.95
Art. anast. (no)	1.5 ± 0.9	1.4 ± 0.8	0.00; 0.13	−0.09	0.032	1.4 ± 0.9	1.4 ± 0.8	−0.06; 0.09	−0.03	0.61
Ven. anast. (no)	1.4 ± 1.0	1.6 ± 1.0	−0.32; −0.17	0.25	<0.01	1.6 ± 1.0	1.6 ± 1.0	−0.11; 0.07	0.02	0.70
Grafts (no)	2.1 ± 0.5	2.2 ± 0.5	−0.10; −0.03	0.13	<0.01	2.2 ± 0.5	2.2 ± 0.5	−0.05; 0.03	0.02	0.74
Art. grafts (no)	1.3 ± 0.5	1.2 ± 0.5	−0.01; 0.07	−0.07	0.07	1.2 ± 0.5	1.2 ± 0.5	−0.05; 0.04	0	>0.99
Ven. grafts (no)	0.8 ± 0.6	0.9 ± 0.5	−0.14; −0.06	0.20	<0.01	0.9 ± 0.5	0.9 ± 0.5	−0.06; 0.03	0.01	0.77

*95% CI* 95% confidence intervals, *AAP* automated anastomosis punching, *ACE* angiotensin-converting enzyme inhibitors, *AHT* arterial hypertension, *Anast.* Anastomosis, *AoSD* aortic sealing device, *AR* aortic regurgitation, *ARB* angiotensin II receptor type 1 blocker, *Art.* Arterial, *AS* aortic stenosis, *ASA* acetylsalicylic acid, *BIMA* bilateral internal mammary artery use, *BMI* body mass index, *CABG* coronary artery bypass grafting, *cAD* central artery disease, *CCB* calcium channel blocker, *CCS* Canadian Cardiovascular Society classification, *COPD* chronic obstructive pulmonary disease, *DM* diabetes mellitus type 2, *EF* left ventricular ejection fraction, *HLP* hyperlipidemia, *LAA* left atrial appendage amputation, *MR* mitral regurgitation, *MRA* mineralocorticoid receptor antagonist, *MS* mitral stenosis, *NYHA* New York Heart Association classification, *OPCAB* off-pump coronary artery bypass grafting, *OR* odds ratio, *pAD* peripheral artery disease, *Preop.* Preoperative, *TAR* total arterial revascularization, *TR* tricuspid regurgitation, *TS* tricuspid stenosis, *Ven.* venous

Parameter estimates are given with their odds ratio (OR), 95% confidence interval (95% CI) and the corresponding *p* value. *p* values <0.05 were considered statistically significant.

## Results

### Cohort

**Fig. 1:**
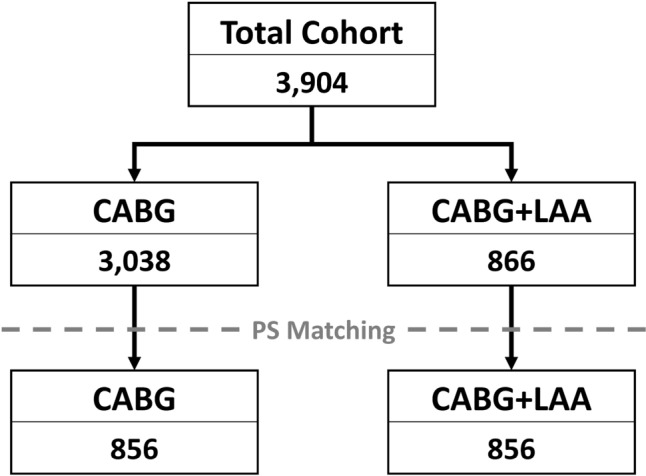
Patient selection process. CABG, coronary artery bypass grafting; LAA, left atrial appendage amputation

A total of 3904 patients who underwent elective coronary artery bypass grafting between 2018 and 2021, were retrospectively included. These patients were divided into the CABG group and the CABG + LAA group with 3038 and 866 patients, respectively. After propensity score (PS) matching, a total of 1712 patients were selected for analysis, with 856 patients in each group (Fig. 1). Before PS matching, 24 baseline characteristics presented a standardized mean difference (SMD) > |0.1| and 27 a *p* value of <0.05, while the matched cohorts showed no variables with significant differences or an SMD > |0.1|. Detailed data regarding the baseline characteristics are summarized in Table 1.

### Primary endpoint

The primary composite endpoint of all-cause mortality, stroke, and reoperation did not show significant differences between the CABG and CABG + LAA cohort with 7.0% vs. 6.5% (OR 0.9 95% CI [0.64; 1.35], *p* = 0.70). The individual components of the primary endpoint all-cause mortality (*p* = 0.84), stroke (*p* = 0.74), and reoperation (*p* = 0.50) did not show any notable differences. The results are summarized in Table 2. Results for the unmatched cohorts are provided in Supplementary Table 1.

**Table 2:** Primary and secondary endpoints in the in-hospital outcome in the CABG and CABG + LAA groups

Variable	CABG *n* (%)	CABG + LAA *n* (%)	OR [95% CI]	*p* value
	856 (100)	856 (100)		
*Primary endpoint*				
Composite endpoint	60 (7.0)	56 (6.5)	0.9 [0.64; 1.35]	0.70
All-cause mortality	13 (1.5)	12 (1.4)	0.9 [0.42; 2.03]	0.84
Stroke	19 (2.2)	17 (2.0)	0.9 [0.46; 1.73]	0.74
Reoperation	38 (4.4)	44 (5.1)	1.2 [0.75; 1.82]	0.50
*Secondary endpoint*				
Need for dialysis	56 (6.5)	37 (4.3)	0.7 [0.42; 0.99]	0.043
Need for ICA	40 (4.7)	35 (4.1)	0.9 [0.55; 1.38]	0.56
IABP	8 (0.9)	5 (0.6)	0.6 [0.20; 1.91]	0.40
ECLS	5 (0.6)	10 (1.2)	2.0 [0.68; 5.91]	0.20
CPR	23 (2.7)	23 (2.7)	1.0 [0.56; 1.80]	>0.99
Pericardial effusion	20 (2.3)	28 (3.3)	1.4 [0.79; 2.53]	0.24
ICU stay (days)	2.0 ± 35.1	0.6 ± 48.9	−2.64; 5.4	0.50
PRBC (units)	2.4 ± 5.1	2.6 ± 7.8	−0.82; 0.4	0.56
FFP (units)	0.8 ± 2.3	0.9 ± 4.4	−0.38; 0.3	0.80
CK_max_ (U/l)	731.4 ± 1063.1	733.3 ± 1131.2	−104.9; 101.2	0.97
CK-MB_max_ (U/l)	−217.9 ± 449.5	−195.6 ± 433.9	−62.5; 17.9	0.28
Troponin_max_ (pg/ml)	8166.1 ± 38,537.1	7364.7 ± 27,639.0	−2390.5; 3993.2	0.62
Creatinine_max_ (mg/dl)	0.4 ± 34.1	1.4 ± 0.9	−3.4; 1.2	0.36

*95% CI* 95% confidence intervals, *CABG* coronary artery bypass grafting, *CK* creatinine kinase, *CK-MB* creatinine kinase isoenzyme MB, *CPR* cardiopulmonary resuscitation, ECLS extracorporeal life support, *FFP* fresh-frozen plasma, *IABP* intra-aortic balloon pump, *ICA* invasive coronary angiography, *ICU* intensive care unit, *LAA* left atrial appendage amputation, *OR* odds ratio, *PRBC* paced red blood cells

### Secondary endpoints

Regarding the secondary endpoints, a significantly higher proportion of patients in the CABG group suffered from acute kidney injury requiring dialysis with rates of 6.5% vs. 4.3% (OR 0.7 95% CI [0.42; 0.99], *p* = 0.043). The groups did not show any differences regarding the other secondary endpoints, neither the occurrence of pericardial effusion (*p* = 0.24), nor CPR (*p* > 0.99) or mechanical circulatory support (IABP (*p* = 0.40), ECLS (*p* = 0.20)). Transfusion rates (PRBC (*p* = 0.56), FFP (*p* = 0.80)) and laboratory parameters (CK_max_ (*p* = 0.97), CK-MB_max_ (*p* = 0.28), Troponin_max_ (*p* = 0.62), Creatinine_max_ (*p* = 0.36)) also did not present relevant variations, either. Furthermore, the patients did not differ in terms of the length of ICU stay (*p* = 0.50) or the need for ICA (*p* = 0.56) (Table 2).

### Subgroup analysis

**Fig. 2:**
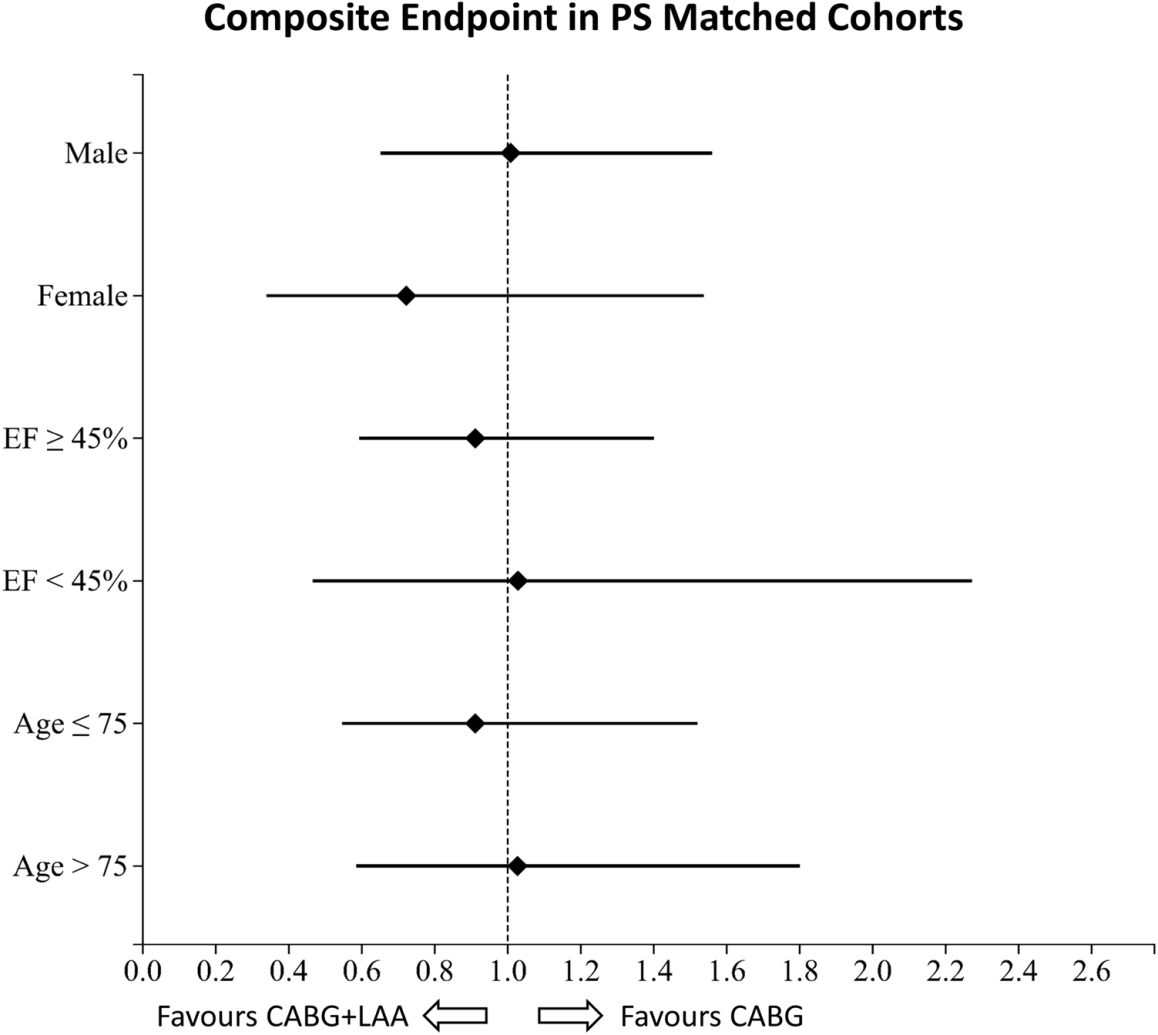
Subgroup analysis of the primary composite endpoint. CABG, coronary artery bypass grafting; EF, left ventricular ejection fraction; LAA, left atrial appendage amputation

Subgroup analysis regarding the primary endpoint did not show any differences in male patients (OR 1.0 95% CI [0.65; 1.56], *p* = 0.97) or in female patients (OR 0.7 95% CI [0.34; 1.54], *p* = 0.40). The subgroup analysis regarding contractility did not show differences in patients with preserved ejection fraction (EF ≥ 45% (OR 0.9 95% CI [0.59; 1.40], *p* = 0.67)) or in patients with reduced ejection fraction (EF < 45% (OR 1.0 95% CI [0.46; 2.27], *p* = 0.95)). In the subgroups based on patient’s age, there were no significant differences in both younger patients (age ≤ 75 years (OR 0.9 95% CI [0.55; 1.52], *p* = 0.72)) and elderly patients (age > 75 years (OR 1.0 95% CI [0.59; 1.80], *p* = 0.93)). The components of the primary composite endpoint did now show any substantial difference in the subgroups. The subgroup analysis is visualized in Fig. 2 and detailed results are provided in Table 3. Results for the unmatched cohorts are given in Supplementary Fig. 1 and Supplementary Table 2.

**Table 3:** Subgroup analysis of the primary composite endpoint and the endpoint composing variables (all-cause mortality, stroke, reoperation)

Variable	CABG *n* (%)	CABG + LAA *n* (%)	OR [95% CI]	*p* value
	856 (100)	856 (100)		
**Gender**				
*Male*	687 (100)	682 (100)		
Composite endpoint	43 (6.3)	43 (6.3)	1.0 [0.65; 1.56]	0.97
All-cause mortality	8 (1.2)	10 (1.5)	1.3 [0.50; 3.22]	0.62
Stroke	16 (2.3)	13 (1.9)	0.8 [0.39; 1.71]	0.59
Reoperation	25 (3.6)	35 (5.1)	1.4 [0.85; 2.42]	0.18
*Female*	169 (100)	174 (100)		
Composite endpoint	17 (10.1)	13 (7.5)	0.7 [0.34; 1.54]	0.40
All-cause mortality	5 (3.0)	2 (1.2)	0.4 [0.07; 1.99]	0.24
Stroke	3 (1.8)	4 (2.3)	1.3 [0.29; 5.91]	0.73
Reoperation	13 (7.7)	9 (5.2)	0.7 [0.27; 1.57]	0.34
**Contractility**				
*EF* ≥ *45%*	707 (100)	721 (100)		
Composite endpoint	46 (6.5)	43 (6.0)	0.9 [0.59; 1.40]	0.67
All-cause mortality	8 (1.1)	7 (1.0)	0.9 [0.31; 2.37]	0.77
Stroke	15 (2.1)	11 (1.5)	0.7 [0.33; 1.57]	0.40
Reoperation	30 (4.2)	35 (4.9)	1.2 [0.70; 1.90]	0.58
*EF* < *45%*	149 (100)	135 (100)		
Composite endpoint	14 (9.4)	13 (9.6)	1.0 [0.46; 2.27]	0.95
All-cause mortality	5 (3.4)	5 (3.7)	1.1 [0.31; 3.91]	0.87
Stroke	4 (2.7)	6 (4.4)	1.7 [0.47; 6.11]	0.42
Reoperation	8 (5.4)	9 (6.7)	1.3 [0.47; 3.36]	0.65
**Age**				
*Age* ≤ *75 years*	540 (100)	590 (100)		
Composite endpoint	31 (5.7)	31 (5.3)	0.9 [0.55; 1.52]	0.72
All-cause mortality	5 (0.9)	8 (1.4)	1.5 [0.48; 4.52]	0.50
Stroke	9 (1.7)	11 (1.9)	1.1 [0.46; 2.73]	0.80
Reoperation	22 (4.1)	24 (4.1)	1.0 [0.55; 1.80]	>0.99
*Age* > *75 years*	316 (100)	266 (100)		
Composite endpoint	29 (9.2)	25 (9.4)	1.0 [0.59; 1.80]	0.93
All-cause mortality	8 (2.5)	4 (1.5)	0.60 [0.18; 1.97]	0.39
Stroke	10 (3.2)	6 (2.3)	0.7 [0.25; 1.97]	0.50
Reoperation	16 (5.1)	20 (7.5)	1.5 [0.77; 3.00]	0.22

*95% CI* 95% confidence intervals, *CABG* coronary artery bypass grafting, *EF* left ventricular ejection fraction, *LAA* left atrial appendage amputation, *OR* odds ratio

## Discussion

This study evaluates the perioperative outcome of left atrial appendage amputation performed concurrently with coronary artery bypass grafting in a retrospective two-center analysis. The results can be summarized into three main points: firstly, there was no difference in the perioperative outcome concerning the composite endpoint of all-cause mortality, stroke and reoperation (I). Secondly, other perioperative outcome parameters showed no relevant differences (II). Finally, subgroups based on gender, myocardial contractility, and age did not indicate any increased risk associated with the procedure (III).

Repeated validation of procedure safety in regard to LAA amputation is crucial, as several studies, most notably the LAAOS III trial [7], have demonstrated the long-term benefit of LAA amputation in reducing stroke risk [7, 14]. These results have strengthened recommendations for the concomitant procedure [8] and are anticipated to result in a significant increase in surgical LAA amputations. However, the translation of results in highly selected cohorts within those trials, must be validated regarding perioperative safety in real-world setting.

The current results demonstrated that patients undergoing LAA amputation concomitant to CABG surgery did not experience worse outcomes. This aligns with findings of several studies which have not shown worse outcomes or longer cardiopulmonary bypass times, but rather a fast-learning curve [7, 15, 16]. In contrast to research indicating sufficient safety of LAA amputation, some research, e.g. Mahmood et al. [17], reported a higher rate of hospital readmissions in patients who underwent LAA amputation, highlighting the need for caution with concomitant procedures and emphasizing the importance to analyze potential complications, subgroups at risk, or procedure associated learning curves.

The continuous demonstration of stroke prevention benefits is of interest not only for concomitant surgical approaches targeting the LAA but also for interventional approaches in patients at elevated risk of bleeding, recurrent stroke, or noncompliance with anticoagulation therapy. The PROTECT AF and PREVAIL trials showed both the safety, but also non-inferiority of interventional LAA occlusion in comparison to oral anticoagulation in patients with AF [18, 19].

Major bleeding itself, a feared postoperative complication in LAA amputation, was not explicitly analyzed in this study. However, it was indirectly assessed through endpoints such as transfusion rate and complications such as CPR, ECLS, IABP as well as the primary endpoint and its components. None of these showed any differences between the CABG and CABG + LAA cohorts. Thus, reoperation was included as part of the primary composite endpoint. Furthermore, it is crucial to point out that even the subclinical occurrence of pericardial effusion did not significantly differ between the assessed groups.

Targeting the LAA may be beneficial for patients with symptomatic atrial fibrillation, by not only preventing stroke but also aiding in rhythm control. The BELIEF trial identified the LAA as a potential trigger of atrial fibrillation [4]. Therefore, amputating the LAA would effectively result in its permanent isolation. The forthcoming results of the ASTRO AF trial (NCT04056390) will be of interest to further showcase the efficacy of LAA isolation.

While LAA amputation is mainly recommended for patients with AF [8], it is necessary to consider the implications for postoperative atrial fibrillation (POAF). POAF commonly occurs after any type of cardiac surgery [20, 21], and even non-cardiac surgery [21, 22], with incidence rates ranging from 20 to 50% [23, 24]. Moreover, it carries a high risk of progressing into long-term AF [25, 26]. Although POAF was not included in the current analysis, no differences in a control cohort and LAA amputation cohort in terms of POAF occurrence or changes in the left atrial size could be demonstrated before [27]. In fact, several studies have demonstrated a significantly impaired outcome in terms of mortality for patients with POAF following cardiac surgery [28, 29]. Therefore, some research considered LAA amputation in patients without a history of AF and identified long-term stroke risk reduction in a 5-year analysis [14], comparable to results of the LAAOS III trial [7], albeit retrospectively. The LeAAPS trial (NCT05478304) will be crucial in clarifying the importance of LAA amputation within this group and in shaping recommendations for LAA amputation in patients with no history of AF. Notably, recent findings indicate that among patients with POAF and LAA amputation, long-term mortality and rehospitalization rates were comparable to those in a control cohort without POAF [30].

When formulating broad recommendations, it is essential to thoroughly understand the affected mechanisms and to identify the subgroups that may benefit the most or face a higher risk of adverse outcomes. Despite the promising long-term results, LAA amputation remains a prophylactic measure and must not interfere with the primary therapeutic goal and result. In this context, the CHA_2_DS_2_-VASc Score serves as a reliable basis [5, 8, 14]. The subgroups analyzed in the current study, visualized in Fig. 2 for gender (male (*p* = 0.97), female (*p* = 0.40)), contractility (EF ≥ 45% (*p* = 0.67), EF < 45% (*p* = 0.95)), and age (≤75 years (*p* = 0.72), >75 years (*p* = 0.93)), present no worse outcomes in any subgroup. This suggests that there are no apparent age- or gender-related differences in tissue stability [31, 32], or age- or contractility-dependent vulnerabilities to concomitant LAA amputation.

## Limitations

Our study has several limitations that warrant consideration. The main limitation of the analysis is its retrospective study design. Detailed information regarding the LAA amputation strategy was not given precluding insights into the still discussed differences of various strategies (clipping, stapling, ligation, amputation) [33]. A *p* value adjustment in subgroup analysis was not performed due to small and underpowered sample sizes predisposing for a type 1 error, yet, a type 2 error cannot be dismissed. The current analysis did only assess “as treated” cohorts with not data regarding the “intention to treat”. Since the execution of LAA amputation mainly depended on the surgeon’s judgment of executability, this introduces potential bias in our cohorts. Preoperative history of AF was not part of the presented baseline parameters, however, the necessary condition to execute concomitant LAA amputation was preoperative atrial fibrillation (paroxysmal, persistent, and permanent). Thus, the number of preoperative AF in the control cohort missing. Perioperative atrial fibrillation was not included in the outcome variables due to missing data inherent to the retrospective nature of the study. Pre- and postoperative anticoagulation were not part of the analysis.

## Conclusion

The concomitant execution of left atrial appendage amputation is not associated with a worse perioperative and in-hospital outcome regarding a composite of all-cause mortality, stroke and reoperation. Subgroup analysis based on gender, contractility, and age also did not reveal any subgroups with a worse outcome.

## Supplementary Information

Below is the link to the electronic supplementary material.

Supplementary file1 (DOCX 1062 KB)

## Data Availability

The data underlying this article will be shared upon reasonable request to the corresponding author.
