# Supplementary material for: Perioperative outcome of left atrial appendage amputation in coronary artery bypass grafting
Source: Clin Res Cardiol. 2024 Sep 2;115(6):916–26. doi: 10.1007/s00392-024-02529-9 (PMC13160959; doi:10.1007/s00392-024-02529-9)
Supplement: Supplementary file 1 — Supplementary file1 (DOCX 1062 KB) [file 392_2024_2529_MOESM1_ESM.docx]

# Supplementary Tables

## Supplementary Table 1 Outcome of POAF and 5-year follow up of all-cause mortality and stroke in patients with preserved and enlarged left atrial diameter in the unmatched cohort

| **Variable** | **CABG**  **n (%)** | **CABG+LAA**  **n (%)** | **OR/HR [95%CI]** | **p-value** |
| --- | --- | --- | --- | --- |
|  | 3,038 (100) | 866 (100) |  |  |
| **Primary Endpoint** | | | | |
| Composite Endpoint | 181 (6.0) | 57 (6.6) | 1.1 [0.82; 1.51] | 0.50 |
| All-Cause Mortality | 29 (1.0) | 12 (1.4) | 1.5 [0.74; 2.87] | 0.27 |
| Stroke | 41 (1.3) | 17 (2.0) | 1.5 [0.83; 2.59] | 0.19 |
| Reoperation | 136 (4.5) | 45 (5.2) | 1.2 [0.83; 1.65] | 0.37 |
| **Secondary Endpoints** | | | | |
| Need for Dialysis | 124 (4.1) | 37 (4.3) | 1.1 [0.72; 1.53] | 0.80 |
| Need for ICA | 121 (4.0) | 36 (4.2) | 1.1 [0.71; 1.53] | 0.82 |
| IABP | 24 (0.8) | 5 (0.6) | 0.7 [0.28; 1.92] | 0.52 |
| ECLS | 20 (0.7) | 10 (1.2) | 1.8 [0.82; 3.78] | 0.14 |
| CPR | 64 (2.1) | 23 (2.7) | 1.3 [0.78; 2.05] | 0.33 |
| Pericardial Effusion | 96 (3.2) | 28 (3.2) | 1.0 [0.67; 1.57] | 0.91 |
| ICU stay [days] | 2.6±6.0 | 2.9±6.7 | -0.83; 0.11 | 0.14 |
| PRBC [units] | 1.8±4.4 | 2.6±7.7 | -1.21; -0.40 | **<0.01** |
| FFP [units} | 0.5±2.0 | 0.9±4.3 | -0.55; -0.15 | **0.024** |
| CK_max_ [U/l] | 897.2±1073.6 | 833.2±1075.1 | -19.27; 147.32 | 0.13 |
| CK-MB_max_ [U/l] | 36.5±69.6 | 35.0±58.5 | -4.30; 7.18 | 0.62 |
| Troponin_max_ [pg/ml] | 4944.6±25269.2 | 7413.8±27606.8 | -4419.17; -519.26 | **0.013** |
| Creatinine_max_ [mg/dl] | 1.3±1.0 | 1.4±0.9 | -0.25; -0.10 | **<0.01** |

Abbreviations: 95%CI, 95% Confidence intervals; CABG, Coronary artery bypass grafting; CK, Creatinine kinase; CK-MB, Creatinine kinase isoenzyme MB; CPR, cardiopulmonary resuscitation; ECLS, Extracorporeal life support; FFP, Fresh-frozen plasma; IABP, intra-aortic balloon pump; ICA, Invasive coronary angiography; ICU, Intensive care unit; LAA, Left atrial appendage amputation; OR, Odds ratio; PRBC, Paced red blood cells.

## Supplementary Table 2 Subgroup analysis (by gender, contractility, age) of the primary composite endpoint and the endpoint composing variables (all-cause mortality, stroke, reoperation) in the unmatched cohort

| **Variable** | | **CABG**  **n (%)** | **CABG+LAA**  **n (%)** | **OR [95%CI]** | **p-value** |
| --- | --- | --- | --- | --- | --- |
|  |  | 3,038 (100) | 866 (100) |  |  |
| **Gender** | **Male** | 2,474 (100) | 687 (100) |  | |
|  | Composite Endpoint | 127 (5.1) | 43 (6.3) | 1.2 [0.86; 1.76] | 0.25 |
|  | All-Cause Mortality | 18 (0.7) | 10 (1.5) | 2.0 [0.93; 4.39] | 0.07 |
|  | Stroke | 30 (1.2) | 13 (1.9) | 1.6 [0.82; 3.03] | 0.17 |
|  | Reoperation | 95 (3.8) | 35 (5.1) | 1.3 [0.90; 2.00] | 0.14 |
|  | **Female** | 564 (100) | 179 (100) |  | |
|  | Composite Endpoint | 54 (9.6) | 14 (7.8) | 0.8 [0.43; 1.48] | 0.48 |
|  | All-Cause Mortality | 11 (2.0) | 2 (1.1) | 0.6 [0.12; 2.59] | 0.46 |
|  | Stroke | 11 (2.0) | 4 (2.2) | 1.2 [0.36; 3.65] | 0.81 |
|  | Reoperation | 41 (7.3) | 10 (5.6) | 0.8 [0.37; 1.54] | 0.44 |
| **Contractility** | **EF ≥ 45 %** | 2,597 (100) | 728 (100) |  | |
|  | Composite Endpoint | 142 (5.5) | 44 (6.0) | 1.1 [0.78; 1.58] | 0.55 |
|  | All-Cause Mortality | 17 (0.7) | 7 (1.0) | 1.5 [0.61; 3.57] | 0.39 |
|  | Stroke | 31 (1.2) | 11 (1.5) | 1.3 [0.64; 2.54] | 0.50 |
|  | Reoperation | 109 (4.2) | 36 (5.0) | 1.2 [0.81; 1.75] | 0.38 |
|  | **EF < 45 %** | 441 (100) | 138 (100) |  | |
|  | Composite Endpoint | 39 (8.8) | 13 (9.4) | 1.1 [0.55; 2.07] | 0.84 |
|  | All-Cause Mortality | 12 (2.7) | 5 (3.6) | 1.3 [0.47; 3.88] | 0.58 |
|  | Stroke | 10 (2.3) | 6 (4.4) | 2.0 [0.70; 5.49] | 0.19 |
|  | Reoperation | 27 (6.1) | 9 (6.5) | 1.1 [0.49; 2.33] | 0.87 |
| **Age** | **Age ≤ 75 years** | 2,368 (100) | 593 (100) |  | |
|  | Composite Endpoint | 123 (5.2) | 32 (5.4) | 1.0 [0.70; 1.55] | 0.84 |
|  | All-Cause Mortality | 16 (0.7) | 8 (1.4) | 2.0 [0.86; 4.72] | 0.10 |
|  | Stroke | 24 (1.0) | 11 (1.9) | 1.9 [0.90; 3.79] | 0.09 |
|  | Reoperation | 100 (4.2) | 25 (4.2) | 1.0 [0.64; 1.56] | 0.99 |
|  | **Age > 75 years** | 670 (100) | 273 (100) |  | |
|  | Composite Endpoint | 58 (8.7) | 25 (9.2) | 1.06 [0.65; 1.74] | 0.81 |
|  | All-Cause Mortality | 13 (1.9) | 4 (1.5) | 0.75 [0.24; 2.33] | 0.62 |
|  | Stroke | 17 (2.5) | 6 (2.2) | 0.86 [0.34; 2.21] | 0.76 |
|  | Reoperation | 36 (5.4) | 20 (7.3) | 1.39 [0.79; 2.45] | 0.25 |

Abbreviations: 95%CI, 95% Confidence intervals; CABG; Coronary artery bypass grafting; EF, Left ventricular ejection fraction; LAA, Left atrial appendage amputation; OR, Odds ratio.

# Supplementary Figures

## Supplementary Figure 1 Subgroup analysis of the primary composite endpoint in the unmatched cohorts


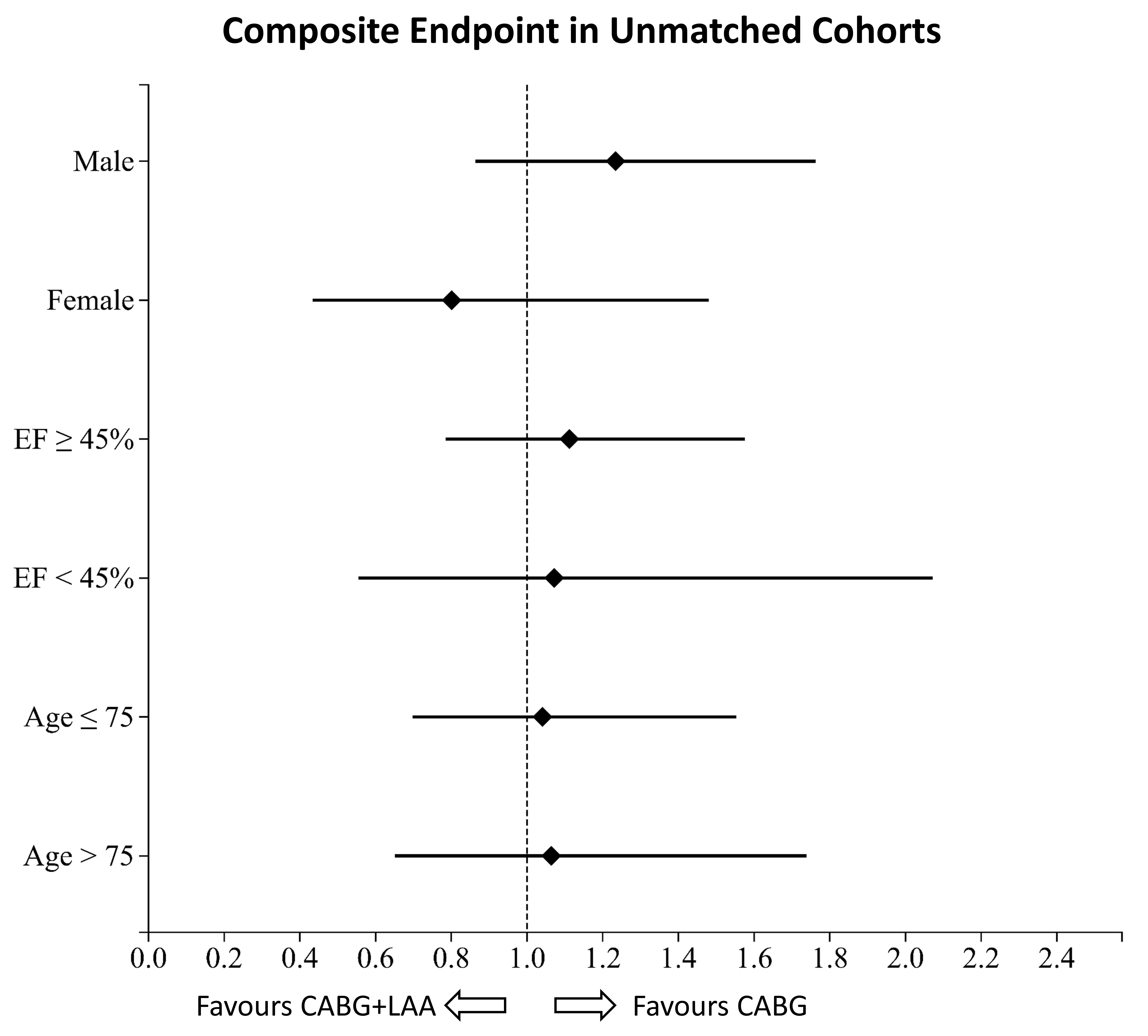


Abbreviations: CABG, Coronary artery bypass grafting; EF, Ejection fraction; LAA, Left atrial appendage amputation.
